# Harnessing ceramic hydroxyapatite as an effective polishing strategy to remove product- and process-related impurities in bispecific antibody purification

**DOI:** 10.1186/s40643-023-00713-9

**Published:** 2023-12-13

**Authors:** Nattha Ingavat, Xinhui Wang, Jia Min Liew, Farouq Bin Mahfut, Ka Pui But, Yee Jiun Kok, Xuezhi Bi, Yuansheng Yang, Kobayashi Shintaro, Maria Tsoumpra, Wei Zhang

**Affiliations:** 1 Downstream Processing Group, Bioprocessing Technology Institute, Agency for Science, Technology and Research (A*STAR), Singapore, Singapore; 2 Cell Line Development Group, Bioprocessing Technology Institute, Agency for Science, Technology and Research (A*STAR), Singapore, Singapore; 3 Protein Analytics Group, Bioprocessing Technology Institute, Agency for Science, Technology and Research (A*STAR), Singapore, Singapore; 4 Chromatography Media Business Division, HOYA Technosurgical Corporation, Singapore Branch, Singapore

**Keywords:** Bispecific antibody, Mixed-mode chromatography, Product- and process-related impurity removal, Chromatography-induced aggregation

## Abstract

Bispecific antibody (bsAb), a novel therapeutic modality, provides excellent treatment efficacy, yet poses numerous challenges to downstream process development, which are mainly due to the intricate diversity of bsAb structures and impurity profiles. Ceramic hydroxyapatite (CHT), a mixed-mode medium, allows proteins to interact with its calcium sites (C-sites) through metal affinity and/or its phosphate sites (P-sites) through cation exchange interactions. This dual-binding capability potentially offers unique bind and elute behaviours for different proteins of interest, resulting in optimal product purity when suitable elution conditions are employed. In this study, the effectiveness of CHT as a polishing step for bsAb purification was investigated across three model molecules and benchmarked against the traditional cation exchange chromatography (CEX). For both asymmetric and symmetric IgG-like bsAb post Protein A eluates, at least 97% product purity was achieved after CHT polishing. CHT delivered a superior aggregate clearance to CEX, resulting in low high molecular weight (HMW) impurities (0.5%) and low process-related impurities in the product pools. Moreover, CHT significantly mitigated "chromatography-induced aggregation" whereas eightfold more HMW was generated by CEX. This study illustrated the developability of CHT in effectively eliminating low molecular weight (LMW) impurities through post-load-wash (PLW) optimization, resulting in an additional reduction of up to 48% in LMW impurities. A mechanistic explanation regarding the performance of impurity removal and mitigation of the chromatography-induced aggregation by CHT was proposed, illustrating unique CHT capability is potentially driven by C-site cooperation, of which effectiveness could depend on the bsAb composition and size.

**Graphical abstract:**

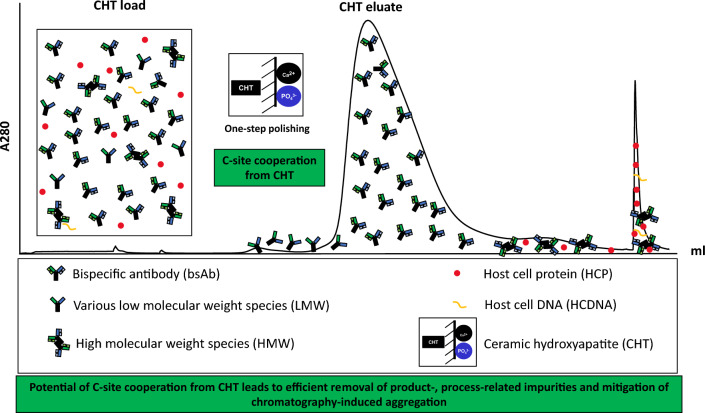

**Supplementary Information:**

The online version contains supplementary material available at 10.1186/s40643-023-00713-9.

## Introduction

Bispecific antibodies (bsAbs) have become one of the most attractive therapeutic modalities over the past decade (Wei et al. 2022; Wu and Cheung 2018). They are man-made proteins, deliberately engineered to possess two different antigen recognition sites instead of one that exists in traditional monoclonal antibodies (mAbs). A combination of the two antigen-binding sites in proximity provides dramatic improvement in therapeutic efficacy (Labrijn et al. 2019). Furthermore, bsAbs can potentially enable novel treatments, not offered by any combination of the natural mAbs.

BsAbs are broadly categorized into three main formats (Chen and Zhang 2021; Labrijn et al. 2019), including fragment-based bsAbs, asymmetric bsAbs, and symmetric bsAbs. The fragment-based bsAbs are designed to incorporate at least two antigen-binding sites into one molecule without the Fc region to avoid any undesired chain association issue (Labrijn et al. 2019). However, this format is likely to have a comparatively short plasma half-life (Labrijn et al. 2019). Both asymmetric bsAbs and symmetric bsAbs are Fc-containing molecules. Asymmetric bsAbs normally contain three to four polypeptides, which are derived from two different parental mAbs. Due to random chain pairing, it is possible for asymmetric bsAbs to generate up to ten different homodimers and heterodimers. Although a couple of bsAb engineering technologies, such as knobs-into-holes (Ridgway et al. 1996; Rouet and Christ 2014) and CrossMAb™ technology (Surowka et al. 2021), have been developed to mitigate chain mispairings, none of these strategies can completely prevent the formation of mispaired byproducts. For symmetric bsAbs, both of their recognition sites are typically incorporated into a single polypeptide chain to allow co-expression of only one to two polypeptides. As a result, they are generally tetravalent (2 + 2) by design. Currently, among all bsAb molecules that have been under clinical development, asymmetric and symmetric IgG-like bsAbs are the most popular formats.

BsAbs pose numerous challenges to downstream processing. The bsAb product-related impurities, including mispaired species, half antibodies, antibody fragments, and aggregates, are difficult to remove because of their similarities in size and physiochemical properties to the desired end-product. Furthermore, engineered bsAbs are also reported to be highly aggregation prone, making them less stable than their parental mAbs (Chen et al. 2022b). Considering the differences between mAb and bsAb, traditional purification tools for mAbs are less likely to provide the same level of efficiency when it comes to bsAbs. In addition, “chromatography-induced aggregation” has been observed during both Protein A and cation exchange chromatography (CEX) for bsAb purification as reported by Serene Chen et al. and Lucus K. Kimerer et al. (Chen et al. 2022a, 2022b; Kimerer et al. 2019). This “chromatography-induced aggregation” is more pronounced when bsAbs are subjected to high loading (Chen et al. 2022a, 2022b). This could be a crucial bottleneck for bsAb industrial production as lower loading leads to lower productivity.

**Fig. 1:**
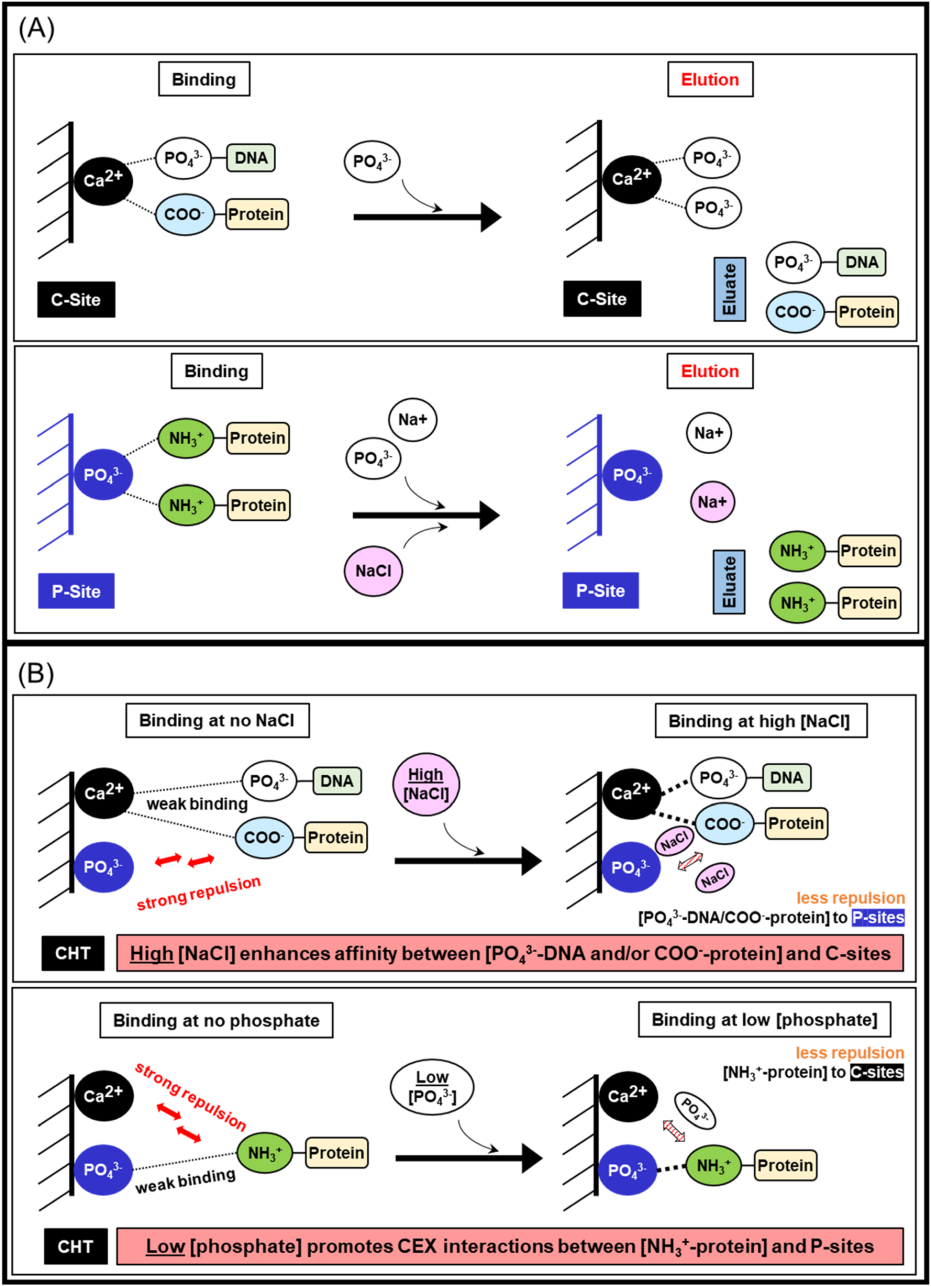
**A** Ceramic hydroxyapatite (CHT) binding (left) and elution (right) mechanisms. There are two types of binding sites on CHT medium, a calcium-site (C-site) (top) and a phosphate-site (P-site) (bottom). **B** The addition of suitable salts (high concentration of NaCl (top); low concentration of phosphate (bottom)) enables stronger binding between biomolecules and the CHT binding sites

Given that ceramic hydroxyapatite (CHT) is one of the leading media for intermediate and polishing step development, it has been applied to the purification of various types of biological therapeutics, including antibodies, antibody fragments, recombinant proteins, viruses, and DNAs (Hilbrig and Freitag 2012). CHT is a mixed-mode support that comprises two different binding sites as illustrated in Fig. 1 A. The first binding site which mainly retains solutes via calcium metal affinity is commonly known as the “C-site”. It primarily binds to carboxyl groups of proteins, phosphoryl residues of DNA, as well as phosphorylated solutes (Gagnon 2009; Itoh et al. 2019). Elution of proteins bound to the C-sites can be achieved with solutions containing either phosphate or fluoride ions, which have higher affinity for calcium ions as compared to carboxyl groups (Gorbunoff and Timasheff 1984). Retention of biomolecules via interactions with the C-sites (e.g. DNA and acidic proteins) can tolerate high concentration of sodium chloride (up to 4 M sodium chloride in the absence of phosphate), suggesting that the C-site interactions are significantly stronger than electrostatic forces (Gagnon 2009; Gorbunoff an Timasheff 1984; Itoh et al. 2019). The second binding site that acts as a cation exchanger is named as the “P-site”. P-sites primarily bind to protonated amino groups on proteins and elution can be accomplished by raising conductivity using any types of salt (Gorbunoff 1984a; Gorbunoff and Timasheff 1984).

Sodium chloride gradient (CHT-NaCl) and phosphate gradients (CHT-P) have been used intensively for CHT elution. In addition, usage of borate and sulphate salts for elution has also been reported for certain applications (Gagnon 2006). Buffer compositions and concentrations play important roles in binding strength, as well as protein binding capacity of CHT. Previous studies showed that sodium chloride can weaken repulsive forces between negatively charged biomolecules (e.g. phosphoryl DNA and carboxyl-proteins) and P-sites. Therefore, interactions of phosphoryl DNA and negatively charged protein with the C-sites are tremendously strengthened at high NaCl concentration (Fig. 1B) (Gagnon 2009; Gagnon et al. 2009; Hilbrig and Freitag 2012). A similar concept applies to interactions between protonated amino groups on proteins and P-sites. During a protein binding state, the presence of low phosphate concentration assists reducing repulsion between protonated amino groups of proteins and the C-sites, as compared to when no phosphate is present. Therefore, strengthening cationic interactions between protonated amino groups of proteins and the P-sites (Gagnon et al. 2009; Gorbunoff 1984b). By tweaking the buffer compositions and concentrations, the binding of the biomolecules to CHT medium can be affected, leading to different binding strength and capacity. In addition, the presence of at least 5 mM phosphate in all buffers is required regardless elution mechanisms to maintain CHT stability (Gagnon 2006, 2009; Gagnon et al. 2009). With a combination of metal affinity and cation exchange interactions, CHT provides unique separation mechanisms with additional purification benefits, therefore improving the removal efficiency of both product- and process-related impurities (Gagnon 2009).

**Fig. 2:**
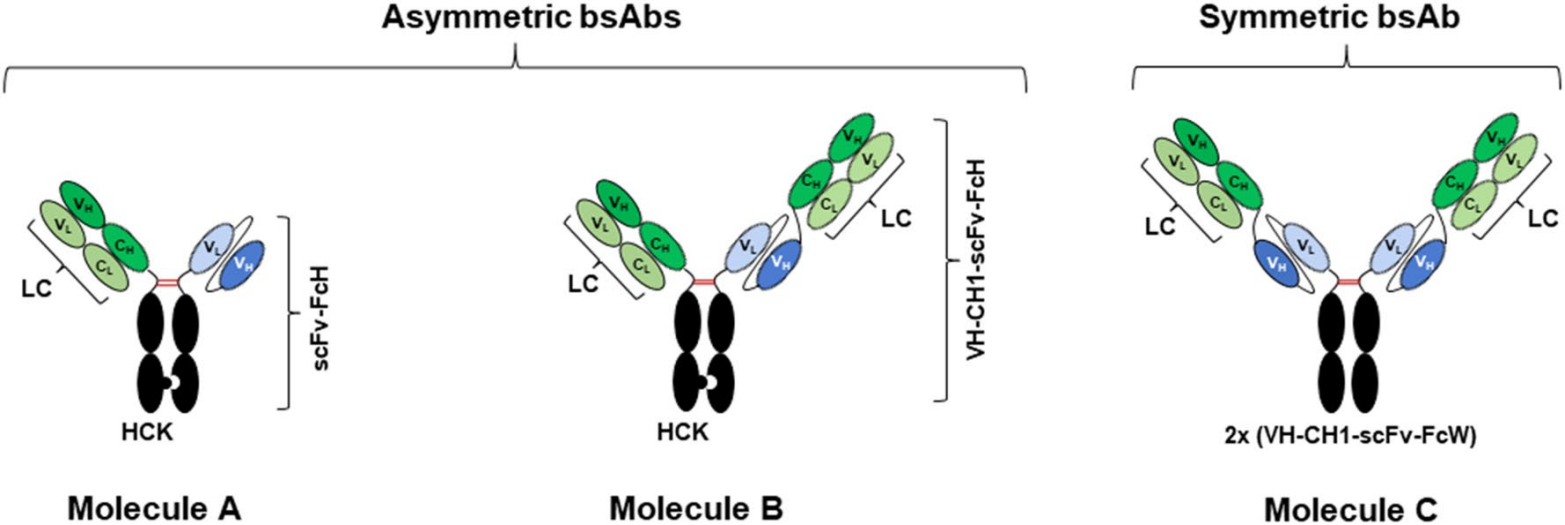
Three bispecific antibodies (bsAbs), used as model molecules in this study, including two asymmetric molecules with heterodimeric knob-in-to-hole Fc and one symmetric molecule with homodimeric wild-type Fc. Molecule A, “1 + 1” valency; Molecule B, “2 + 1” valency; Molecule C, “2 + 2” valency. Abbreviations: LC = Light chain; HCK = Heavy chain knob; scFv-FcH = scFv-Fc hole; VH-CH1-scFv-FcH = VH-CH1-scFv-Fc hole; and VH-CH1-scFv-FcW = VH-CH1-scFv-Fc wild type

In this study, the effectiveness of CHT chromatography as a polishing step on bsAb purification was evaluated and compared to the traditional CEX, using three model bsAb molecules (Molecules A, B, and C) with different formats, molecular sizes, and domain compositions as illustrated in Fig. 2. The results showed that CHT chromatography can serve as an effective polishing step for both asymmetric and symmetric IgG-like bsAbs, interestingly, with different binding and elution mechanisms. The products of both bsAb formats were of 97% purity with acceptable levels of process-related impurities. Previously, Yiran Wang, et al. have conducted mechanistic studies on competitive binding kinetics and separation dynamics of monomeric and dimeric monoclonal antibodies using CHT (Wang and Carta 2019, 2020a). A hybrid model has also been developed to explain separation of monoclonal antibody monomer–dimer mixture using gradient elution strategies (Wang and Carta 2020b). On top of the previous published papers, herein, we extended CHT application to bsAb purification and elaborated the possible mechanistic insights. Our mechanistic explanation mainly focuses on how CHT medium mediates both product- and process-related impurity removal in bsAb purification.

## Materials and methods

### Materials

All buffers, salts, and reagents were purchased from Sigma-Aldrich except for sodium chloride and citric acid that were purchased from Merck Millipore. MabSelect PrismA, Capto SP ImpRes, and Tricon series columns were purchased from Cytiva. CHT Type II (40 µm) was provided by HOYA Technosurgical Corporation. CHT Type II (40 µm) is manufactured by HOYA Technosurgical Corporation and distributed globally by Bio-Rad Laboratories, Inc. Hercules, CA, USA.

### BsAb culture production

Stably transfected CHO K1 cell lines, producing bsAbs, were generated by site-specific integration of plasmid vectors, which carried genes encoding light chain (LC), heavy chain knob (HCK) and scFv-Fc hole (scFv-FcH) (for Molecule A), or VH-CH1-scFv-Fc hole (VH-CH1-scFv-FcH) (for Molecule B); LC and VH-CH1-scFv-Fc wild type (VH-CH1-scFv-FcW) (for Molecule C) (Fig. 2). CH3 domains in the HCK and scFv-FcH/VH-CH1-scFv-FcH were engineered to form a knob (through mutations of S354C:T366W) and a hole (through mutations of Y349C:T366S:Y407V), respectively, to facilitate heterodimeric Fc pairing, based on a previous study (Merchant et al. 1998). For Molecule A, VH and VL in scFv were connected through a flexible linker (G4S)3, which was further linked to FcH through a G4 linker. The engineered Fc consisted of IgG1 positions 221 to 447 based on the Eu numbering system (Wang et al. 2018). For Molecules B and C, VH-CH1 was linked to the N-terminus of scFv through a G4S linker, which was further linked to either FcH (Molecule B) or FcW (Molecule C), respectively, through a G4 linker. The stably transfected cell lines were grown in a protein-free medium consisting of HyQ PF (Cytiva) and CD CHO (Thermo Fisher Scientific) at 1:1 ratio and supplemented with 1 g/L sodium carbonate (Sigma), 6 mM glutamine (Sigma), and 0.1% Pluronic F-68 (Thermo Fisher Scientific) in 50 mL tubespin (TPP) in a humidified Kuhner shaker (Adolf Kühner AG) with 8% CO_2_ at 37 °C. To produce these three molecules, 300 mL of cell culture at viable cell density of 3 × 10^5^ cells/mL was inoculated into 600 mL tubespin (TPP) in the humidified Kuhner shaker (Adolf Kühner AG) with 8% CO_2_ at 37 °C. 30 mL of Ex-Cell Advanced CHO Feed 1 (with glucose) (SAFC, Sigma) was added at days 3, 5, 7, 9, and 11. Cell density and viability of each culture were monitored at days 3, 5, 7, 9, 11, and 14, using the Vi-Cell XR viability analyser (Beckman Coulter). d-Glucose concentration in the culture medium was quantified using Nova BioProfile 100plus Analyzer (Nova Biomedical). When the glucose concentration in the media dropped below 2 g/L, d-glucose (Sigma) was added to the culture to adjust glucose concentration above 6 g/L. Culture supernatant was harvested at day 14, then centrifuged and filtrated to remove cells and cell debris before proceeding to purification.

### ÄKTA™ chromatography

BsAb harvest cell culture fluids (HCCFs) were purified using MabSelect PrismA resin to obtain post Protein A products, which were adjusted to pH 6.5, using 1 M Tris-base. The neutralized bsAb samples were then aliquoted and frozen at – 20 °C. These samples were used for the polishing step study. Prior to CEX/CHT purification, the frozen bsAb samples were thawed at room temperature, then adjusted to pH 5.5 for CEX load and pH 6.8 for CHT load, all conductivities were kept ≤ 5 mS/cm.

1 mL of CHT Type II medium (Bio-Rad) and Capto SP ImpRes resin (Cytiva) was packed in Tricorn™ series columns (Cytiva), respectively, with a bed height of 5.1 cm. Purifications were conducted using AKTA™ Avant 25 (Cytiva) with a flow rate of 150 cm/h unless otherwise stated. Eluates were collected as fractions (1 mL/fraction) into a 96-well deep well plate. Fractions with higher purity than loading material were mock pooled with equal volume and re-analysed by HPLC-SEC to determine yield and purity.

For CHT evaluation runs, the packed column was pre-equilibrated with 500 mM sodium phosphate pH 6.8 (3 CV), then equilibrated with equilibration buffer (10 mM sodium phosphate pH 6.8) (10 CV) prior to loading (10 ± 1 mg protein/mL medium; monomer concentration of 1 ± 0.1 mg/mL). After loading, a post-load-wash (PLW) was conducted using the equilibration buffer (5 CV). Linear gradient elution was performed using either 10–400 mM sodium phosphate pH 6.8 (for phosphate gradient elution) or 10 mM sodium phosphate pH 6.8 with 0–400 mM sodium chloride (for sodium chloride gradient elution), both over 40 CV with additional 10 CV hold at 400 mM phosphate or sodium chloride concentration. For optimized CHT sodium chloride purification, PLW was modified by using 45 mM or 60 mM sodium phosphate pH 6.8 (10 CV, flowrate = 75 cm/h) for Molecules A and B, respectively, followed by 10 mM sodium phosphate pH 6.8 (3 CV, flowrate = 75 cm/h) before proceeding to the same linear sodium chloride gradient elution profile as performed in the scouting runs.

For CEX evaluation runs, the column was pre-equilibrated with 50 mM sodium acetate pH 5.5, and 1 M sodium chloride (5 CV) then equilibrated with an equilibration buffer (50 mM sodium acetate pH 5.5) (5 CV) prior to loading (10 ± 1 mg protein/mL resin; monomer concentration of 1 ± 0.1 mg/mL), being followed by a PLW using the equilibration buffer (5 CV). Linear gradient elution was performed using 50 mM sodium acetate pH 5.5 with 0–400 mM sodium chloride over 40 CV with 10 CV additional hold at 400 mM sodium chloride concentration.

### BsAb concentration and purity determination

BsAb concentration and purity analysis were performed by size exclusion chromatography-HPLC (HPLC-SEC), using a TSKgel G3000SWXL column (7.8 mm i.d. × 30 cm; Tosoh Bioscience), flow rate: 0.6 mL/min; mobile phase: 200 mM L-arginine, 50 mM MES, 5 mM EDTA, 0.05% sodium azide (w/w), pH 6.5, UV 280 nm. The determination of bsAb concentrations was achieved by measuring the area under the main peak and referencing a calibration curve generated with known concentrations of antibody standards (Chen et al. 2022b). In the HCCF, the main peak may potentially include other impurities, such as specific HCPs. To ensure accurate bsAb monomeric quantification, we applied a correction method by subtracting the integration of the main peak from the HCCF from that of the flowthrough obtained from PrismA chromatography. This effectively eliminated background interference from the calculation of bsAb concentration. The quantities of HMW and LMW components were determined by analysing the peak areas eluting before and after the main peak, respectively.

SDS-PAGE gels (Bio-Rad, non-reducing Gel: 4–15% Criterion TGX Stain-Free Protein Gel, reducing Gel: Any KD Criterion TGX Stain-Free Protein Gel) were used to visualize target proteins and impurities. 0.2 µg of monomeric bsAb was loaded per lane. The gels were stained using eLuminol™ (GeneCopoeia).

### Residual host cell protein (HCP) and host cell DNA (HCDNA) determination

CHO HCP contents were determined by CHO HCP ELISA Kit, 3G (Cygnus Technologies). The protocol was executed, following the manufacturer’s instructions. Data acquisition was performed on the Synergy™ 2 plate reader (BioTek).

CHO HCDNA contents were determined using a qPCR assay. Briefly, all bsAb samples were digested with proteinase K (0.2 mg/mL in 0.5% SDS, 1 h, 50 °C), followed by heat inactivation (10 min, 95 °C) and DNA extraction using QIAamp® viral RNA mini kit (Qiagen). HCDNA contents were then determined from the extracted samples, using resDNASEQ Quantitative CHO DNA Kit (Thermo Fisher Scientific), following the manufacturer’s instructions. Data acquisition was performed on LightCycler 96 (Roche).

### Intact mass analysis by LC–MS

Purified bsAb samples were diluted with 0.3% formic acid, and 200 ng of each sample was separated on nanoACQUITY UPLC (Waters) with Waters BioResolve RP mAb Polyphenyl Column (450 Å, 2.7 µm, 1 mm X 150 mm) at a flow rate of 50 µL/min, with 2 min desalting, 6 min gradient from 20 to 90% mobile phase (0.1% formic acid in acetonitrile), followed by 4 min equilibration of 20% mobile phase. Protein intact mass data were acquired using TripleTOF 6600 mass spectrometry (SCIEX) in a positive ion mode at 600–3500 m/z, spray voltage of 5000 V, source temperature of 400 °C, and operated by Analyst 1.8. MS data were processed and deconvoluted using Byos 4.3 Intact Mass™ software (Protein Metrics Inc).

## Results

### Purification media evaluation for polishing asymmetric and symmetric IgG-like bsAbs

The performance of CHT for bsAb polishing was evaluated in comparison to CEX resin. Notably, there are two major variations of CHT, Type I and Type II, each possessing distinctive characteristics. CHT Type I exhibits a higher dynamic binding capacity, particularly for acidic proteins. In contrast, CHT Type II, while offering a relatively lower binding capacity, excels in providing enhanced resolution for nucleic acids and certain other proteins. Given that the bsAb molecules under investigation in this study all have basic isoelectric points (pI), CHT Type II was selected as the medium of choice (Bio-Rad; Carta 2018; Wang & Carta 2020a). Capto SP ImpRes was chosen as the CEX resin for comparison with CHT because it is a popular purification medium, established for polishing mAbs industrially (Luo et al. 2014; Sharkey et al. 2017). In addition, Capto SP ImpRes possesses a comparable particle size to that of the CHT Type II (40 µm) (BioRad; Cytiva 2020). As CHT is a mixed-mode medium, runs with CHT-NaCl and CHT-P were performed to explore both elution mechanisms. As a result, a total of nine purification runs were conducted on the model bsAbs to evaluate the performance of CHT and CEX as a polishing step.

**Fig. 3:**
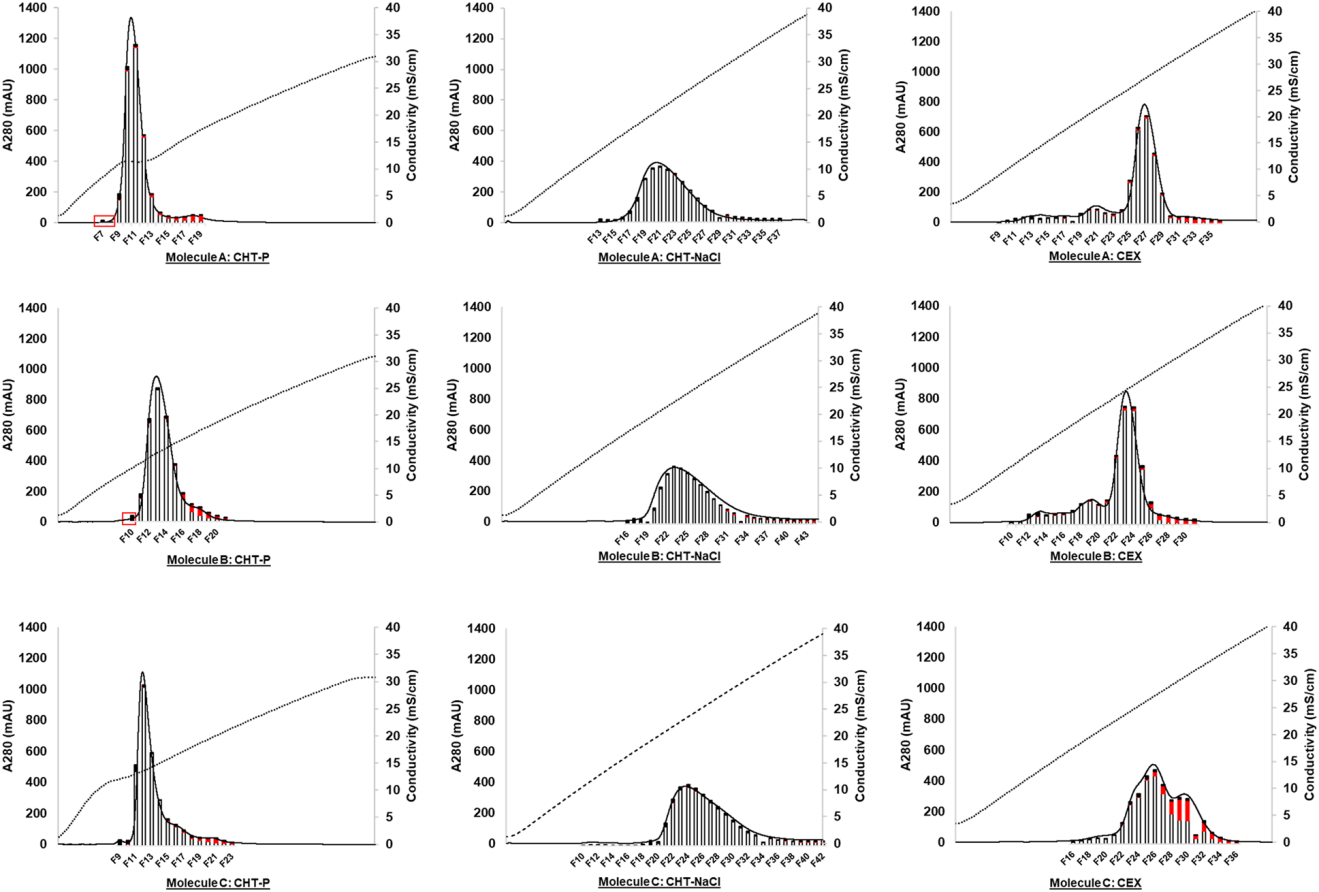
Elution profiles of Molecule A (top); Molecule B (middle); and Molecule C (bottom) from CHT phosphate gradient elution (CHT-P, left column); CHT-NaCl gradient elution (CHT-NaCl, middle column); and CEX (right column). Each plot demonstrates an overlay of chromatogram (A280) with protein mass in each elution fraction (bar graph), comprising Main (white); HMW (red); and LMW (black). The x-axis demonstrates volume during an elution phase (40 CV). Dotted lines demonstrate conductivity (mS/cm), required for corresponding elution fractions. Red boxes indicate elution fractions, containing mainly LMW species

**Fig. 4:**
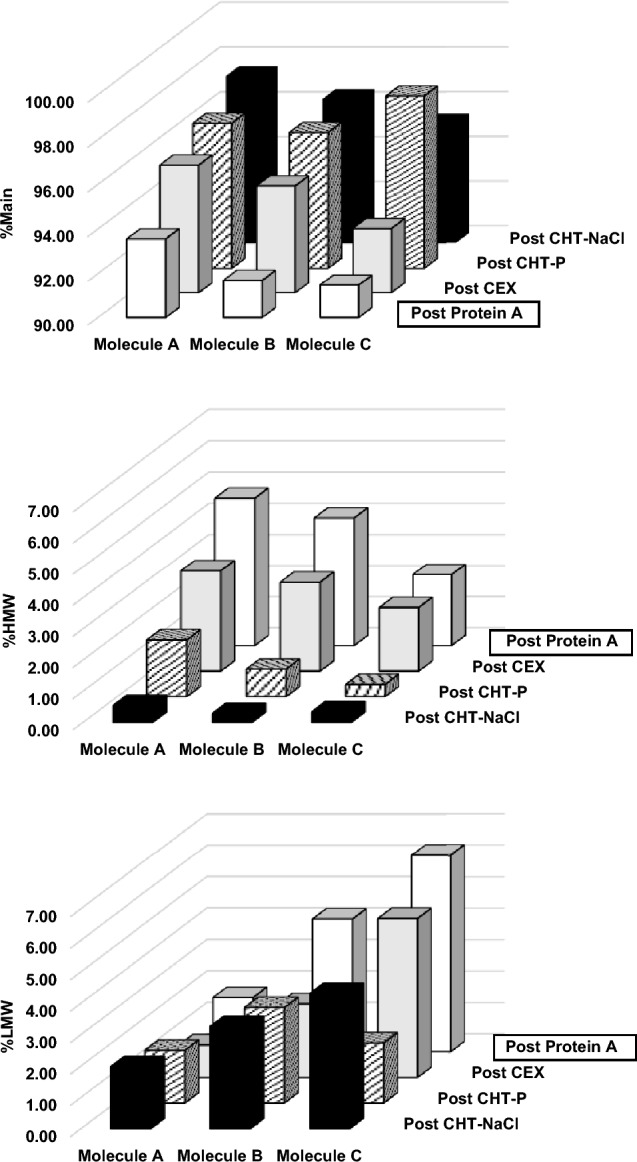
Bar graphs, demonstrating product purity (%Main (top); %HMW (middle); %LMW (bottom) of Molecules A, B, and C after each chromatography step (post Protein A, post CEX, post CHT phosphate gradient elution (CHT-P), and post CHT sodium chloride gradient elution (CHT-NaCl))

Gradient elution profiles of the nine purification runs are illustrated in Fig. 3. The results revealed that CHT chromatography resulted in higher product purity for all three bsAbs, compared to CEX chromatography. The highest monomer purity for both asymmetric bsAbs (Molecules A and B) was obtained from CHT-NaCl runs (%Main = 97.5% (Molecule A); and 96.5% (Molecule B)). Instead, a symmetric bsAb (Molecule C) achieved the best monomer purity of 97.7% from the CHT-P run (Fig. 4 and Table 1). As such, CHT-NaCl and CHT-P were chosen for asymmetric and symmetric bsAbs purification, respectively.

**Table 1:** Product purity (%), HCP and HCDNA contents in loads, and product pools of Molecules A, B, and C after Protein A, CEX, CHT-P, and CHT-NaCl chromatography

Molecule	Protein samples	Purity (%)			HCP (ppm)	HCDNA (ppm)
		HMW	Main	LMW		
A	HCCF	21.32	35.39	43.29	1,012,636	100,223
	Post Protein A	4.73	93.55	1.72	5839	39
	Post CEX	3.23	95.74	1.03	301	0.008
	Post CHT-P	1.82	96.49	1.68	378	N.D
	Post CHT-NaCl	0.53	97.49	1.98	73	N.D
	Post CHT-NaCl (optimized-PLW)	0.47	98.49	1.04	62	0.009
B	HCCF	30.98	34.18	34.84	391,286	51,832
	Post Protein A	4.10	91.69	4.22	4578	17
	Post CEX	2.85	94.81	2.34	43	0.006
	Post CHT-P	0.88	96.08	3.04	155	N.D
	Post CHT-NaCl	0.28	96.46	3.26	34	N.D
	Post CHT-NaCl (optimized-PLW)	0.15	97.36	2.50	12	N.D
C	HCCF	25.16	30.97	43.87	380,062	108,858
	Post Protein A	2.28	91.47	6.25	1304	7
	Post CEX	2.04	92.90	5.05	243	0.026
	Post CHT-P	0.39	97.70	1.91	70	N.D
	Post CHT-NaCl	0.33	95.36	4.31	35	0.012

Indicated as N.D. means the amount was not detectable

More efficient aggregate removal for all three bsAbs was observed through the employment of CHT chromatography, compared to CEX. For asymmetric bsAbs, post CEX product pools still contained up to 3.2% HMW species, while as low as 0.3% HMW was present in post CHT products. CHT-NaCl showed higher efficiency in removing HMW species than CHT-P, with approximately threefold less HMW contents, detected in post CHT-NaCl products for both Molecules A and B. Interestingly, the HMW removal efficiency by CHT-NaCl and CHT-P became more convergent for the symmetric bsAb Molecule C (%HMW = 0.39% (CHT-P) and 0.33% (CHT-NaCl)), suggesting merely equivalent performance for aggregate removal by both CHT-P and CHT-NaCl.

**Fig. 5:**
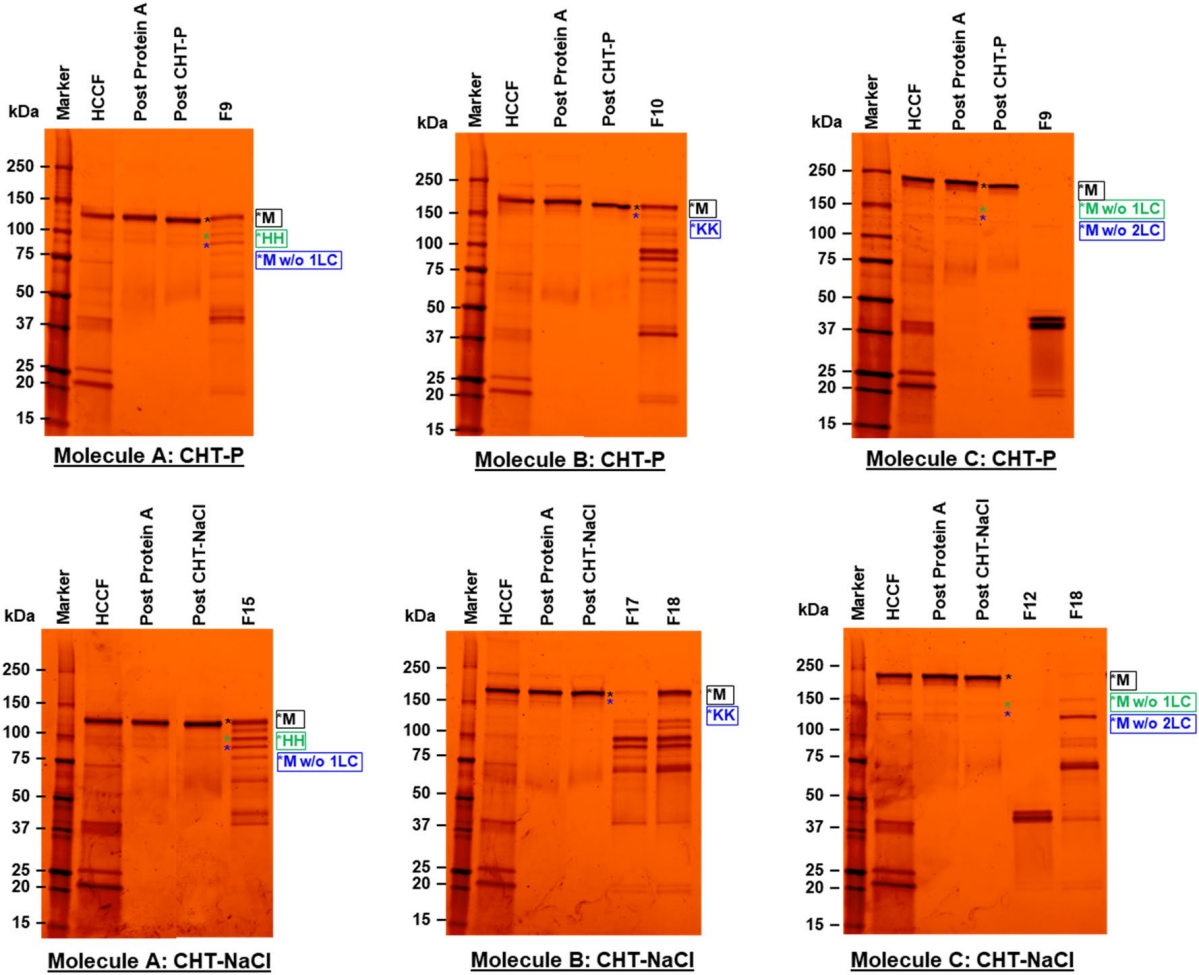
Non-reducing SDS-PAGEs, illustrating protein populations in HCCF, Post Protein A, Post CHT-P (top), Post CHT-NaCl (bottom), and early elution fractions prior to main product peaks (Fx) of Molecule A (left column); Molecule B (middle column); and Molecule C (right column), respectively. Marker units are in kDa. Protein populations, indicated as (*), were proposed as suggested by molecular weights, which were calculated from amino acid sequences. Abbreviations used are listed as follows; M = monomer; HH = hole–hole homodimer; KK = knob–knob homodimer; LC = light chain; w/o = without. Existence of large LMWs in early elution fractions indicated early elution of large LMWs, as compared to monomers

Regarding LMW removal, small LMW species, such as light chain monomers (23.4 kDa) and dimers (46.8 kDa), could be fully removed by all tested chromatography runs as evidenced by SDS-PAGE, showing no light chain co-eluted with the products (Fig. 5, data not shown for CEX). However, larger LMW species, including hole–hole homodimer (103.6 kDa) and monomer without one light chain (101.1 kDa) in Molecule A; knob–knob homodimer (145.6 kDa) in Molecule B; and monomers, lacking one or two light chains (153.0 and 176.4 kDa, respectively) in Molecule C, were still co-eluted with the targets in high-purity elution fractions. As evidenced by SDS-PAGE that the light chain monomers and dimers were completely removed from the products, the LMW species, being detected by SEC-HPLC, were primarily larger LMW species. The SEC-HPLC results indicated that CEX offered the best large LMW removal for asymmetric bsAbs Molecules A and B, followed by CHT-P runs that did slightly better than the CHT-NaCl runs (Molecule A: %LMW = 1.0% (CEX); 1.7% (CHT-P); 2.0% (CHT-NaCl); and Molecule B: %LMW = 2.34% (CEX); 3.04% (CHT-P); 3.26% (CHT-NaCl)) (Fig. 3, Fig. 4, and Table 1). Nevertheless, the best large LMW removal performance was achieved by CHT-P in symmetric bsAb Molecule C (%LMW = 1.9% (CHT-P); 4.3% (CHT-NaCl); 5.1% (CEX)).

**Fig. 6:**
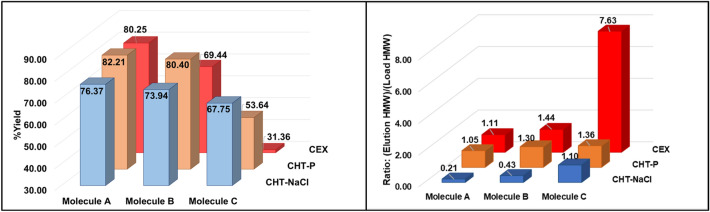
Bar graphs, illustrating %yield (left); and “chromatography-induced aggregation (right) of Molecules A, B, and C after CHT sodium chloride gradient elution (CHT-NaCl), CHT phosphate gradient elution (CHT-P), and CEX. Levels of “chromatography-induced aggregation”, was defined by the ratios of [HMW contents in all elution fractions] over [HMW contents in loads]. HMW contents in the fractionated elutes during the gradient elution and that in CEX and CHT loads were calculated based on SEC-HPLC analysis to generate the ratios for evaluation. Higher ratios indicated more aggregates were generated during chromatography

Product yields across different medium/elution mechanisms were also evaluated and compared within the same molecule (Fig. 6 (left)). %Yield of asymmetric bsAbs Molecules A and B across all purification runs are generally comparable (Molecule A: %yield = 76.4% (CHT-NaCl); 82.2% (CHT-P); 80.3% (CEX); Molecule B: %yield = 73.9% (CHT-NaCl); 80.4% (CHT-P); 69.4% (CEX)) (Fig. 6 (left)). However, %yield for Molecule C was relatively lower than that of Molecules A and B. This was potentially because Molecule C is the most aggregation-prone molecule among all tested bsAbs. The monomers could have been associated to form aggregates on the medium during purification, thus leading to lower monomer yield. %Yield of the symmetric bsAb Molecule C was significantly improved when using CHT chromatography as CHT provided higher separation resolution than that of the CEX (%yield = 67.8% (CHT-NaCl); 53.6% (CHT-P); 31.4% (CEX)) (Fig. 6 (left)). In addition, CHT caused much less “chromatography-induced aggregation” (Fig. 6 (right)).

One of the most challenging issues, reported for bsAb purification, is so-called “chromatography-induced aggregation” (Chen et al. 2022a, 2022b). This phenomenon is where monomeric bsAbs are self-associated during chromatographic purification, resulting in more HMWs in the product pools, together with reduction in monomer yield. With similarly low monomeric concentrations of both loading materials (1 mg/mL) and eluates (CEX = 0.6–0.9 mg/mL; CHT-NaCl = 0.6–0.8 mg/mL; CHT-P = 1.3–2.6 mg/mL), aggregation propensity across all molecules and chromatographic runs could potentially be compared. Herein, we defined ratios of [HMW contents in all elution fractions] over [HMW contents in loads] to represent degrees of “chromatography-induced aggregation”. The higher ratios indicate that more HMWs are generated during purification. Our study showed that less “chromatography-induced aggregation” was detected in CHT runs as compared to CEX runs (Fig. 6 (right)). For both asymmetric bsAbs, up to ~ fivefold more HMWs were generated during CEX chromatography than those observed during CHT runs. The effect was more exaggerated for the symmetric bsAb, Molecule C, where almost eightfold more aggregates were formed during CEX purification, while the “chromatography-induced aggregates” were barely detected in post CHT products. These results implied the symmetric Molecule C expressed much higher aggregation propensity than both asymmetric bsAbs, but the CHT medium could impressively mitigate aggregation formation during the process, while CEX was unable to.

## Developability of CHT purification methods to enhance LMW removal

**Fig. 7:**
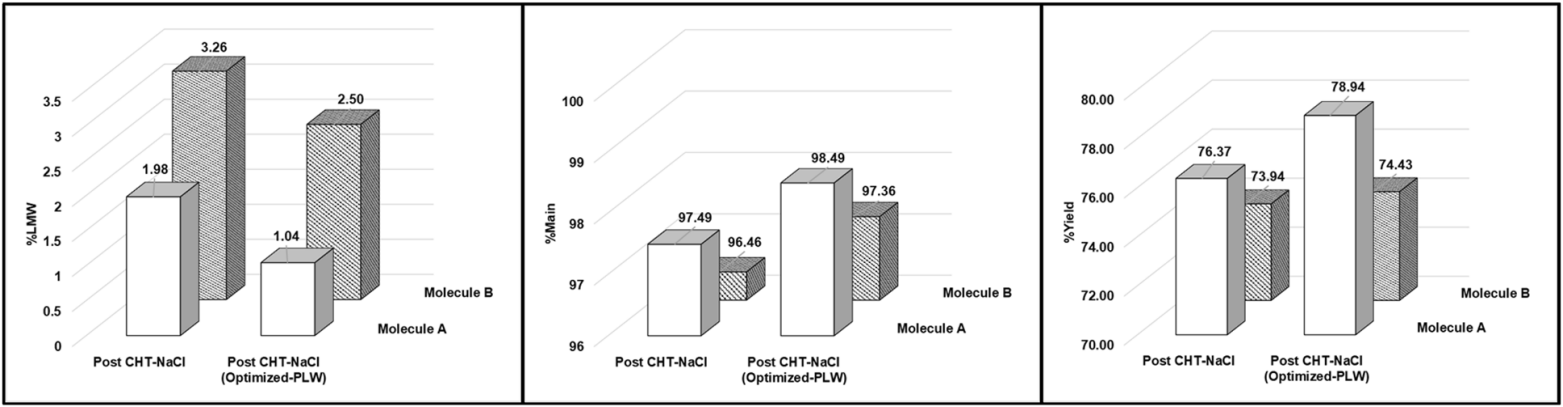
Bar graphs, showing %LMW (left); %Main (middle); and %Yield (right)) of Molecule A, and B post CHT-NaCl vs post CHT-NaCl (optimized PLW)

Since post CHT-NaCl eluates of asymmetric bsAbs Molecules A and B still contained higher levels of LMWs, comparing to those from CEX-NaCl runs, optimization of CHT-NaCl runs was conducted to evaluate the developability of CHT purification methods for further LMW removal. As shown in Fig. 3 (indicated by red boxes), LMW species could be removed from Molecules A and B at elevated phosphate concentrations (> 10 mM, used during the media evaluation study) with minimal loss of monomers. Therefore, a modified post-load-wash (PLW) step with slightly higher phosphate concentration was implemented for both asymmetric bsAbs. Phosphate concentrations were increased from 10 to 45 mM for Molecule A, and to 60 mM for Molecule B, respectively. The CVs of PLW were also extended from 5 to 10 CV, and the wash flow rate was reduced by half to potentially improve LMW removal efficiency. Modifications on the PLW step led to further reduction of LMW contents in product pools by 48% for Molecule A, and 23% for Molecule B, as compared to the CHT-NaCl evaluation run (Fig. 7 (left)). Consequently, product purity was improved from 97.5% to 98.5% for Molecule A, and from 96.5% to 97.4% for Molecule B (Fig. 7 (middle)). Although some larger LMW species still co-eluted with the target proteins (Additional file 1: Figure S1), over 97% purity is typically considered as acceptable for bsAb products (Li et al. 2020; Tustian et al. 2016). Expectedly, improvement upon separation resolution led to slight increase in %yield for both asymmetric bsAb Molecules A and B (%yield from 76.4% to 78.9% for Molecule A, and from 73.9% to 74.4% for Molecule B, respectively) (Fig. 7 (right)).

## Determination of HCP and HCDNA contents

Residual CHO HCP and HCDNA contents were evaluated on the load and product from each purification step (Table 1). As expected, reduction of these process-related impurities after each purification step was observed with depletion of HCDNA contents to undetectable levels across all tested polishing media and elution mechanisms (Table 1). However, the amount of HCP present in product pools after each chromatography run differed. Interestingly, high-purity products from CHT-NaCl consistently expressed minimal HCP contents (HCP = 12 – 73 ppm), whereas those from both CEX and CHT-P showed higher levels of HCP contaminants (HCP = 43 – 378 ppm) (Table 1). The results suggested that the CHT-NaCl has unique mechanisms to efficiently modulate HCP removal from the product pools.

## Characterization of purified bsAbs

Intact mass analysis confirmed that identities of all three bsAb molecules were conserved after each chromatography step (Protein A, CEX, and CHT) (Additional file 1: Figures S2–4). For Molecule C, CEX purification showed two dominant elution peaks (Fig. 3), both of which were confirmed to be the target molecule (Additional file 1: Figure S4).

## Discussion

### Higher conductivity requirements in CHT-NaCl vs. CHT-P Gradient Elution

Conductivity, required for bsAb to be eluted out from CHT medium, was higher with CHT-NaCl gradient elution than with CHT-P gradient elution (Fig. 3), suggesting that bsAb binding to CHT medium was stronger during NaCl gradient elution in comparison with that of the phosphate gradient. This phenomenon could be the outcome of C-site cooperation, which behaved differently during CHT-NaCl vs CHT-P elution.

**Fig. 8:**
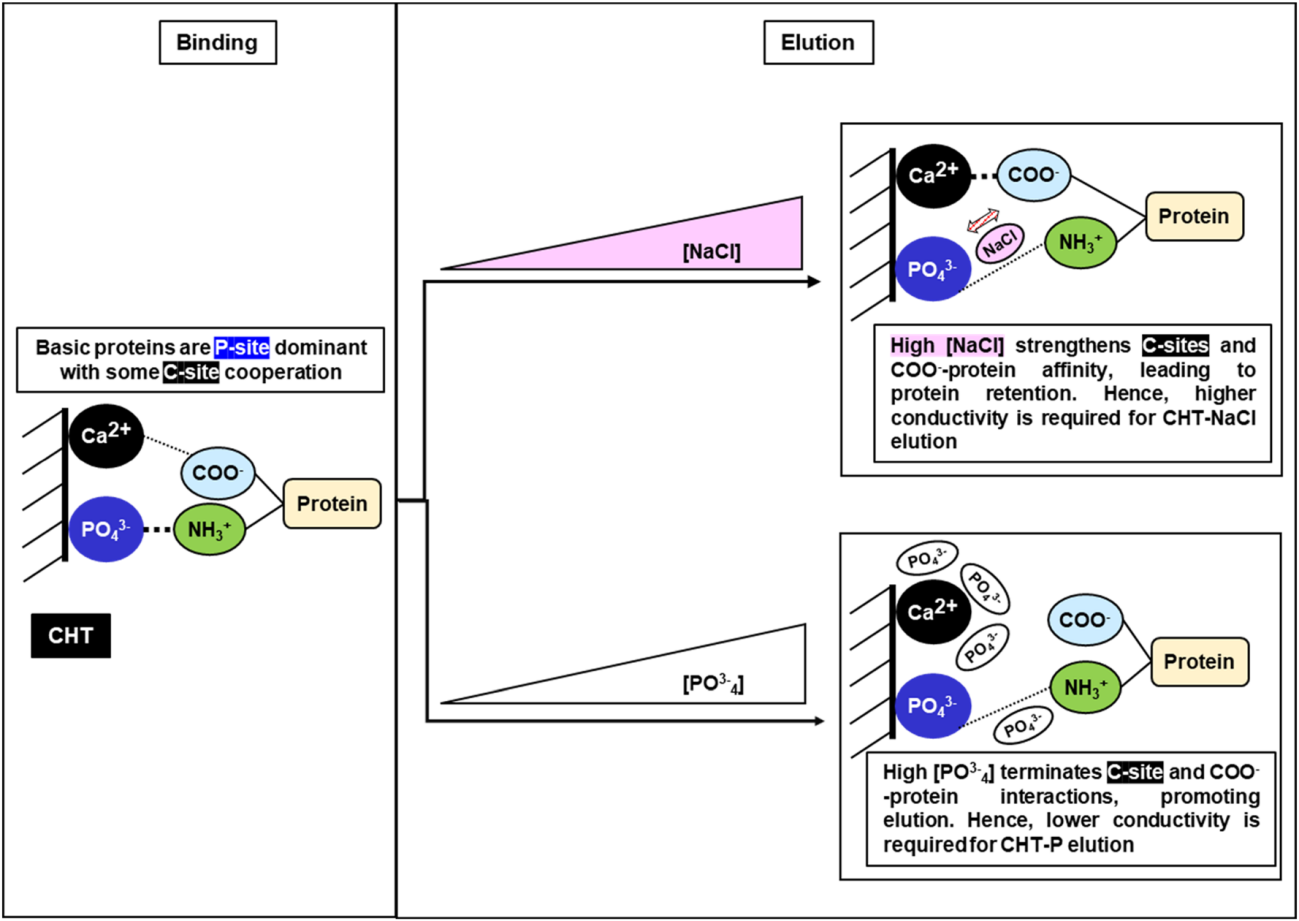
Binding (left) and elution mechanisms for CHT-NaCl (top right) and CHT-P (bottom right)

Figure 8 illustrates the possible differences in binding and elution mechanisms for CHT-NaCl and CHT-P. Even though Molecules A, B, and C binding to CHT medium was P-site dominant as indicated by pI their pI values (pI = 8.52 (Molecule A); 8.59 (Molecule B); 8.70 (Molecule C), parts of the protein surfaces could still interact with C-sites via carboxyl clusters (Gorbunoff 1984b). Prior to elution, bsAb binding to CHT medium was at the same state with the same strength in both CHT-NaCl and CHT-P runs, as the same loading and washing conditions were applied. When the CHT-NaCl gradient started, higher conductivity weakened the electrostatic interactions between P-sites and protonated amino groups on proteins, which served as the main driving force for bsAb elution from CHT medium. However, higher NaCl concentration also enhanced C-site interactions to carboxyl groups on protein surfaces, which concurrently leading to further protein retention on CHT. Consequently, higher elution conductivity was required for CHT-NaCl elution due to C-site cooperation. When it came to CHT-P, the increase of phosphate concentration gradually saturated C-sites and led to the termination of C-site cooperation, which facilitated bsAb elution from CHT. This explains why lower conductivity for elution was required for CHT-P, as compared to CHT-NaCl. This evidence demonstrated that buffer composition and concentration have direct impacts on protein-CHT binding, either strengthening or weakening the binding strength.

### Positive cooperation from C-sites enhanced CHT performance for product-related impurity removal

#### Efficient HMW removal by CHT

CHT chromatography proved to offer better aggregate removal than CEX for all three tested bsAbs (Molecules A, B, and C), and CHT-NaCl showed the best performance Fig. 3, Table 1). For Molecules A and B, CHT-NaCl significantly outperformed CHT-P, as evidenced by approximately threefold less HMWs detected in post CHT-NaCl product. However, CHT-P showed almost equivalent HMW removal efficiency to CHT-NaCl for Molecule C, as closer HMW contents were detected in both post CHT-P and CHT-NaCl products (%HMW = 0.39% (CHT-P); and 0.33% (CHT-NaCl). The results implied CHT-NaCl has a distinctive mechanism to improve separation resolution, and molecular size might be a key factor to promote HMW removal efficiency by CHT-P for Molecule C (MW = 124.6 kDa (Molecule A); = 172.5 kDa (Molecule B); = 199.8 kDa (Molecule C)).

For Molecules A and B, better HMW removal by CHT-NaCl could be simply explained by the different C-site cooperation during CHT-NaCl and CHT-P elution as well. Aggregates are significantly larger than monomers in size and have higher surface charges. They had stronger interactions to both P-sites and C-sites on CHT, thus requiring higher conductivity for elution. During CHT-NaCl gradient elution, higher conductivity triggered protein elution but at the same time high NaCl concentration also strengthened affinity between C-sites and carboxyl groups on protein surfaces (Fig. 8, top right). Since aggregates were larger than monomers, this C-site retention effect was stronger towards aggregates than monomers, thus leading to even higher conductivity required for aggregates elution. The difference in C-site cooperation between aggregates and monomers during elution enhanced their separation resolution. However, such mechanism was not applicable when performing CHT-P elution. Our observation is also supported by the previous work, demonstrating that a monomeric IgG and tetra-aggregates were eluted at significantly different NaCl concentrations when using CHT-NaCl (Gagnon et al. 2009). Interestingly, HMW removal performance by CHT-P was on par with that of CHT-NaCl for Molecule C, of which the molecular size is larger than both Molecules A and B due to the presence of two flexible scFv domains (Fig. 2). This was potentially because Molecule C had stronger initial binding strength to both P-sites and C-sites due to its larger protein surface, especially for aggregates. Therefore, the difference in binding strength between aggregates and monomers was significant enough to provide good separation resolution during elution, no matter CHT-NaCl or CHT-P was applied. This evidence implied the “molecular size” could be a notable factor, accounting for different binding and separation behaviours. As proven by our study, CHT-P could also yield great HMW removal efficiency to the same level as provided by CHT-NaCl when it came to larger bsAbs Molecule C. A similar observation was reported in previous studies, where CHT-P was successfully applied to purify large monoclonal antibody types, like IgA (385 kDa) and IgM (900 kDa) (Aoyama & Chiba 1993; Gagnon 2009; Luellau et al. 1998; Lüllau et al. 1997). Obviously, CEX separation was solely achieved by the difference in electrostatic interactions between aggregates and monomers. As such, HMW removal by CEX was lower than that of both CHT-P and CHT-NaCl.

#### Efficient LMW removal by CHT

Various LMW populations existed in post Protein A for all three bsAbs (Molecules A, B, and C), which could be broadly categorized into small and large LMW species (Table 2.). Light chain monomers and dimers could be efficiently removed by both CHT and CEX chromatography as they were small, hence containing lesser binding surfaces and net charges, as indicated by low pI values (Table 2.). This left mainly larger LMWs to co-elute with the target monomers. Interestingly, different media and elution mechanisms demonstrated different performance on large LMW removal.

**Table 2:** Structures, Molecular weights (MW), pI values, Fc (%), and ∆Fc (%) of bsAb Molecules A, B, and C for monomers and LMW species

Molecule	Category	Protein species	Structure	MW (kDa)	pI Value	Fc (%)	∆Fc (%)
A, B, C	Small LMWs	LC-M		23.4	7.76	0%	-40%^A^, -29%^B^, and -26%^C^
		LC-D		46.9	7.98	0%	
A	Target	M		124.6	8.52	40%	0%
	Large LMWs	HH		103.6	8.64	48%	8%
		M—LC		101.2	8.58	49%	9%
B	Target	M		172.5	8.59	29%	0%
	Large LMWs	KK		145.5	8.43	35%	6%
C	Target	M		199.8	8.70	26%	0%
	Large LMWs	M—LC		176.4	8.74	29%	3%
		M—2LC		153.0	8.80	33%	8%

MW values were calculated based on amino acid sequences. pI values were calculated from an Expasy ProtParam tool. Fc (%) and ∆Fc(%) are as defined in Eq. 2 and Eq. 3, respectively. Abbreviations used are listed as followed; D = dimer, M = monomer, HH = hole–hole homodimer; KK = knob–knob homodimer; LC = light chain. ∆Fc (%)A, ∆Fc (%)B, and ∆Fc (%)C of LC-M and LC-D are as compared to monomers of Molecules A, B, C, respectively. The more positive ∆Fc (%) between product-related impurities and monomers may suggest higher effectiveness of impurity removal via CHT-P elution

By comparing two CHT elution mechanisms, CHT-P always provided better large LMW removal than CHT-NaCl for all tested bsAbs, yet to different extents (Table 1). Better separation resolution by CHT-P suggested that monomers and large LMW impurities expressed divergent binding preference to CHT binding sites, where ones could be more favoured to C-sites than the others. Herein, we defined “C-site binding ratios” (Eq. 1) to evaluate on CHT binding site preference for three domain compositions of bsAb Molecules A, B, and C (scFv, Fab, and Fc), then identified which domain binding was the most favoured to C-sites. To simplify the calculations, only amino acids, giving major contributions to C-site (Asp and Glu) and P-site (Arg and Lys) binding, were considered. A protein domain with a higher C-site binding ratio was supposed to be more desirable to bind to C-sites. As illustrated in Table 3, Fc domains (Fc (knob), Fc(hole), and Fc (wild type)) owned higher C-site binding ratios than both scFv and Fab domains. Hence, Fc domains would favourably bind to C-sites, while binding of both scFv and Fab had higher tendency towards P-sites. This was also inline with the pI values (Table 3). In addition, our rationale was supported by the previous empirical study, explaining different domain compositions of IgGs demonstrated different preferences to CHT binding sites, where binding of Fab and (Fab)2 regions is P-site dominant but binding of a Fc domain is more favoured to the C-sites (Gagnon et al. 2009).

**Table 3:** C-site binding ratios, pI values, and C-site binding preference for scFv, Fab, Fc domain compositions for bsAbs Molecules A, B, and C

bsAb Domain	C-site binding ratio	pI value	C-site binding preference
scFv	0.70	9.09	Less favoured
Fab	0.85	8.64	
Fc (knob)	1.0	6.94	More favoured
Fc (hole)	1.0	6.94	
Fc (wild type)	1.0	7.18	

Fc (knob) and Fc (hole) are Fc domains of Molecules A and B, while Fc (wild type) is the Fc domain for Molecule C

We then further investigated on domain compositions of larger LMWs and monomers for bsAb Molecules A, B, and C, and defined Fc(%) (Eq. 2) and ∆Fc(%) (Eq. 3) (see Appendices) to explain our results in a mechanistic point of view. Table 2 illustrates that large LMW impurities for all three bsAbs (Molecules A, B, and C) contained higher percentage of the Fc region (Fc (%)) than their monomers (positive ∆Fc(%)). Therefore, these large LMWs were likely to have more preferential binding towards the C-sites, as compared to the monomers.

**Fig. 9:**
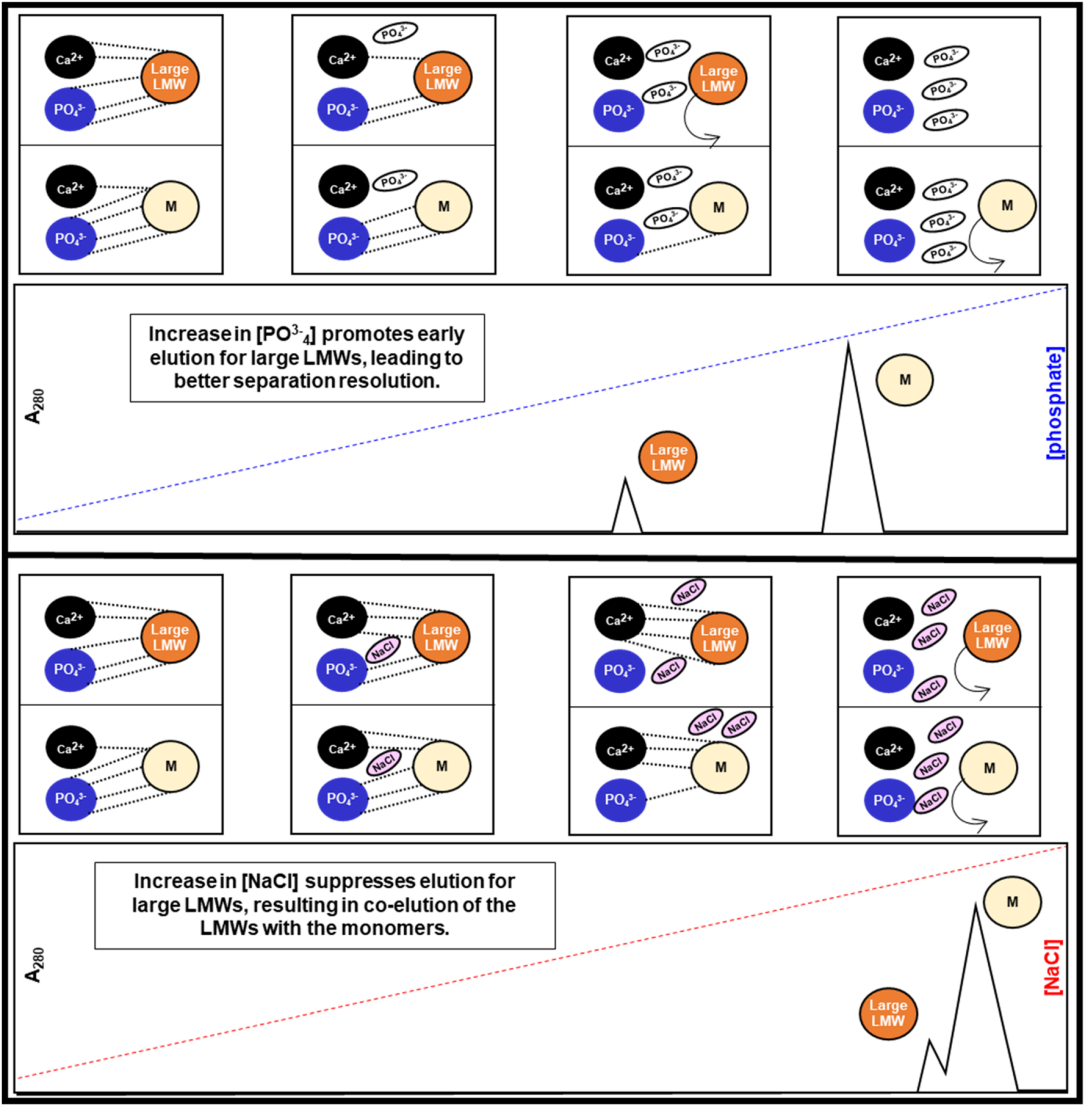
Mechanistic illustrations during CHT-P (top) and CHT-NaCl (bottom) gradient elution. Differences in interactions between large LMWs vs monomers to C- and P-sites on CHT resulted in better separation resolution by CHT-P over CHT-NaCl. Numbers of dotted lines, NaCl and phosphate molecules are used for an illustration purpose to compare protein-CHT binding behaviour (from left to right) during CHT-NaCl and CHT-P gradient elution, but not quantitatively

Greater large LMW removal efficiency by CHT-P than CHT-NaCl seemed to also be driven by the positive cooperation from C-sites. As shown in Fig. 9, during CHT-P gradient elution, gradual increase in amounts of phosphate could trigger elution of large LMWs prior to monomer elution (Fig. 5, Additional file 1: Figure S5) as those impurities expressed more C-site cooperation. While those impurities were eluting, monomers, which had stronger electrostatic interactions, were still hold mainly on P-sites, leading to higher separation resolution by CHT-P. Instead, during CHT-NaCl gradient, hiking in NaCl concentration strengthened protein affinity to C-sites, leading to further retention of both impurities and the target monomers. Once proper conductivity was achieved, large LMWs were simply co-eluted with the target monomers, resulting in lower separation efficiency by CHT-NaCl as compared to CHT-P.

Interestingly, CHT-P provided just slightly better large LMW removal than CHT-NaCl for Molecules A and B (Molecule A: %LMW = 1.7% (CHT-P); 2.0% (CHT-NaCl); Molecule B: %LMW = 3.0% (CHT-P); 3.3% (CHT-NaCl)), while the difference was more pronounced for Molecule C (%LMW = 1.9% (CHT-P); 4.3% (CHT-NaCl)), and this can be potentially attributed to the difference in their molecular sizes. As bsAb Molecules A and B were smaller than Molecule C, their concurrent binding with both C- and P-sites on CHT was potentially less pronounced than Molecule C. Thus, C-site cooperation was more beneficial for larger protein like Molecule C, enabling higher separation resolution between large LMWs and the target monomers.

The C-site cooperation also well explained better large LMW separation by CEX than CHT-NaCl for Molecules A and B. As CEX did not have C-sites to retard elution of large LMWs at high NaCl concentration, LMW species were likely to elute earlier by CEX than CHT-NaCl, resulting in better separation resolution. Impressively, CHT-P could even outperform CEX for Molecule C to remove large LMW species. This evidence was also driven by larger molecular size of Molecule C, allowing the protein to make the most use of C-site cooperation for the best performance on LMW removal efficiency among all tested chromatographic runs.

Based on our findings, we generally concluded that differences in C-site binding preferences, coupled with molecular size considerations, significantly influence CHT separation performance in various bsAbs. It is intriguing to explore how structural variations in bsAbs interact with CHT ligands, offering opportunities for customized CHT optimization based on specific structural and domain composition. However, bsAb structures are complex and highly variable, making in-depth understanding challenging. Advanced technique like in silico modelling is necessary for a comprehensive investigation (Hou et al. 2011), but this approach requires data from a large protein library which is beyond the scope of our current study.

### CHT-NaCl showed great performance on process-related impurity removal

Consistently low HCP contents were observed in post CHT-NaCl products for all three bsAbs (Table 1), indicating that the sodium chloride elution mechanism can efficiently remove HCPs from the product pools. Other previous studies also demonstrated similar observations (Gagnon 2009). This could be explained by pI values of HCPs, along with purification process conditions. The CHO HCPs have a wide range of pI values (pI = 2–11), majority of which are between pI values of 4.5–7.0 (Chollangi et al. 2015). After a neutralization step (post Protein A, at pH of 6.5), some HCPs with neutral pI values could potentially be eliminated via precipitation, leaving majority of the acidic HCPs in CHT and CEX loads. As acidic proteins were well coordinated with the C-sites on the CHT medium, products eluted via sodium chloride gradient contained less HCPs. This was because those acidic HCPs still bound to the C-sites during elution with sodium chloride, whereas application of phosphate solutes totally abolished this acidic HCP holding mechanism. Obviously, the CEX chromatography had no such mechanism to hold these acidic HCPs, resulting in co-elution of the acidic HCPs with the target proteins.

Although HCDNA contents in both post CHT and post CEX products were undetectable in our study (Table 1), other previous work demonstrated that CHT-NaCl provided not only lower HCP contents than CHT-P performed, but it also yielded lower contents of other process-related impurities, including HCDNA and endotoxins (Gagnon 2009).

### CHT-NaCl and CHT-P significantly mitigated “chromatography-induced aggregation” against highly aggregation-prone bsAbs

In general, bsAbs are highly aggregation-prone proteins, especially for the scFv-containing molecules (Chen et al. 2022a, 2022b; Li et al. 2016). Chromatography-induced aggregation for both Molecules A and B have been previously reported, when performing a bind–elute mode for both protein A and CEX chromatography (Chen et al. 2022a, 2022b). Our study further reaffirmed the phenomenon. In addition, we observed that symmetric bsAb Molecule C is more prone to aggregation than the other two asymmetric bsAbs Molecules A and B. This is potentially because Molecule C contains two scFv domains, which are known to be less folded, more hydrophobic and with higher aggregation propensity (Fig. 2).

One of the most impactful advantages of CHT is that it mitigated aggregate formation during purification, while significantly higher aggregate contents were generated during CEX chromatography (Fig. 6 (right)). A possible mechanistic explanation for minimal aggregate formation by CHT is likely due to the presence of calcium ions at C-sites. During column chromatography, proteins are bound on the medium at high concentrations, making intermolecular interactions more favourable than intramolecular interactions (Baek and Zydney 2018). Thus, more aggregate formation could be expected during the purification process given hydrophobic interactions as key driving forces. Chaotropic salts, such as calcium chloride and arginine hydrochloride, have been commonly used as additives to suppress “chromatography-induced aggregation” in protein purification (Chen et al. 2020; Luo et al. 2014; Song et al. 2023). (Chen et al. 2020; Luo et al. 2014; Song et al. 2023). Given this evidence, our hypothesis posits that calcium structures installed at the C-sites on CHT may also contribute to alleviating the issue as the immobilized calcium ions are likely to disrupt hydrogen bonding networks of water, which potentially reduces solution polarity, in other words, increasing hydrophobicity in the solution (Jacob 2000). This alteration in solution characteristics may provide additional sources of hydrophobic interactions with proteins. This ultimately diminishes intermolecular hydrophobic interactions among protein molecules, resulting in reduced protein aggregation (Pham and Meng 2020). This phenomenon potentially explained why CHT could mitigate “on-column aggregation”, whereas CEX lacked this capability. The potential of CHT to minimize process-induced aggregates accentuates its suitability for bsAb purification in a commercial scale to achieve high productivity.

Different strategies were explored previously to enhance aggregate removal in bsAb purification by CEX, among which the use of elevated alkaline pH during elution seemed to be promising, as demonstrated by pH 8.1 for elution (Hall et al. 2015). However, this method only benefited bsAb molecules with strong alkaline tolerance which limited its application. In addition, split peaks were commonly observed during CEX elution (Fig. 3) (Carta 2018) which could possibly be attributed to chromatography-induced conformational changes and reversible self-association, potentially leading to bsAb aggregation. In contrast, minimal split peaks were shown in CHT elution profiles under the same elution conditions (Fig. 3). Our study evidenced the significant reduction of chromatography-induced aggregation in CHT compared to CEX, rendering it particularly valuable for polishing bsAbs which are known for their high aggregation propensity. CHT also demonstrated remarkable efficiency in the removal of HCPs and HCDNA. The above evidence underscores CHT as a superior choice for bsAb polishing.

## Conclusion

Our study demonstrated the effectiveness of CHT Type II medium in bsAb purification, covering both asymmetric and symmetric IgG-like bispecific formats, providing the products with at least 97% purity. CHT chromatography consistently achieved remarkable aggregate removal, with CHT-NaCl outperforming both CEX and CHT-P in general. Importantly, “chromatography-induced aggregation” was rarely detected in post-CHT products, especially in CHT-NaCl runs. Furthermore, CHT-NaCl introduced a unique mechanism to effectively remove HCP. Based on the results in this study, we propose customized elution strategies to enhance product purity for different bsAbs. CHT-NaCl is potentially a promising choice for bsAbs similar in size to IgG (e.g., Molecules A and B) to achieve optimal HMW removal. For larger bsAbs (e.g., Molecule C), CHT-P elution is suitable to potentially remove both HMW and large LMW impurities. The myriad benefits of CHT mentioned above are highly likely attributed to its C-site cooperation.

While CHT chromatography has certain limitations that require attention, there are effective approaches to overcome them. Specifically, in cases where the removal of large LMW species in bsAbs, similar in size to IgG (e.g., Molecules A and B) poses a challenge, a combination of CHT-NaCl with Protein A chromatography using a low pH PLW strategy can enhance the removal of large LMW species, including half antibodies and homodimers. (Chen et al. 2022b). Although CHT-P was less effective to remove process-related contaminants (e.g. HCPs and HCDNA), this elution mechanism can still be utilized, mainly for larger and more complex bsAb molecules (e.g. Molecule C) to remove challenging large LMW and aggregates. An orthogonal polishing strategy (e.g. anion exchange chromatography (Li 2017)) can always be coupled with CHT-P to further remove the process-related impurities in order to meet drug safety compliance. To implement our findings at an industrial scale, future studies should investigate dynamic binding capacity, develop step elution strategies, and facilitate process scaling for practical industry use.

### Supplementary Information

**Additional file 1: Figure S1.** Non-reducing SDS-PAGEs, illustrating protein populations in HCCF, Post Protein A, Post CHT-P (optimized-PLW), and elution fractions before main product peaks (Molecule A: F16) or Molecule B: PLW fraction, of Molecules A and B, respectively. Marker units are in kDa. Protein populations, indicated as (*), were proposed as suggested by molecular weights, which were calculated from amino acid sequences. Abbreviations used are listed as followed; M = monomer; HH = hole–hole homodimer; KK = knob–knob homodimer; LC = light chain; w/o = without. **Figure S2.** Intact mass analysis of Molecule A; post Protein A eluate; post CEX main elution peak; post CEX product pool; and post CHT-NaCl product pool with PLW optimization, respectively. **Figure S3.** Intact mass analysis of Molecule B; post Protein A eluate; post CEX main elution peak; post CEX product pool; and post CHT-NaCl product pool with PLW optimization, respectively. **Figure S4.** Intact mass analysis of Molecule C; post Protein A eluate; post CEX main elution peak1; post CEX main elution peak2; post CEX product pool; and post CHT-P product pool, respectively. **Figure S5.** SEC-HPLC chromatograms of F9 (early elution fraction and F15 (monomer elution fraction) from a CHT-P run for the symmetric bsAb Molecule C. The chromatogram showed that CHT-P was able to remove large LMW species prior to elution of the target monomer.

## Data Availability

All data generated or analysed during this study are included in this published article [and its additional information files].

## Appendices

$$C-site binding ratio= \frac{\left[\# of Asp+Glu\right]}{\left[\# of Arg+Lys\right]}.$$

Equation 1. Calculation of C-site binding ratio

$$Fc\left(\%\right)= \frac{\left(MW of Fc regions\right)}{(\mathrm{MW of full molecule})}\times 100.$$

Equation 2. Fc(%) Calculation

$$\Delta {\text{Fc}}\left(\mathrm{\%}\right)=\left({\text{Fc}}(\mathrm{\%})\mathrm{ of the fragment of interest}\right)-\left({\text{Fc}}\left(\mathrm{\%}\right)\mathrm{of monomer}\right).$$

Equation 3. ∆Fc(%) Calculation.

## Abbreviations

bsAbs: Bispecific antibodies
CEX: Cation exchange chromatography
CHT: Ceramic hydroxyapatite
CHT-NaCl: Ceramic hydroxyapatite with sodium chloride linear gradient elution
CHT-P: Ceramic hydroxyapatite with sodium phosphate linear gradient elution
HCCF: Harvest cell culture fluid
HCP: Host cell proteins
HMW: High molecular weight
LMW: Low molecular weight
mAbs: Monoclonal antibodies
PLW: Post-load-wash
