# Supplementary material for: Harnessing ceramic hydroxyapatite as an effective polishing strategy to remove product- and process-related impurities in bispecific antibody purification
Source: Bioresour Bioprocess. 2023 Dec 13;10(1):93. doi: 10.1186/s40643-023-00713-9 (PMC10992335; doi:10.1186/s40643-023-00713-9)
Supplement: Supplementary file 1 — Additional file 1: Figure S1. Non-reducing SDS-PAGEs, illustrating protein populations in HCCF, Post Protein A, Post CHT-P (optimized-PLW), and elution fractions before main product peaks (Molecule A: F16) or Molecule B: PLW fraction, of Molecules A and B, respectively. Marker units are in kDa. Protein populations, indicated as (*), were proposed as suggested by molecular weights, which were calculated from amino acid sequences. Abbreviations used are listed as followed; M = monomer; HH = hole–hole homodimer; KK = knob–knob homodimer; LC = light chain; w/o = without. Figure S2. Intact mass analysis of Molecule A; post Protein A eluate; post CEX main elution peak; post CEX product pool; and post CHT-NaCl product pool with PLW optimization, respectively. Figure S3. Intact mass analysis of Molecule B; post Protein A eluate; post CEX main elution peak; post CEX product pool; and post CHT-NaCl product pool with PLW optimization, respectively. Figure S4. Intact mass analysis of Molecule C; post Protein A eluate; post CEX main elution peak1; post CEX main elution peak2; post CEX product pool; and post CHT-P product pool, respectively. Figure S5. SEC-HPLC chromatograms of F9 (early elution fraction and F15 (monomer elution fraction) from a CHT-P run for the symmetric bsAb Molecule C. The chromatogram showed that CHT-P was able to remove large LMW species prior to elution of the target monomer. [file 40643_2023_713_MOESM1_ESM.docx]

**Additional material**

**Harnessing Ceramic Hydroxyapatite as an Effective Polishing Strategy to Remove Product- and Process-related Impurities in Bispecific Antibody Purification**

Nattha Ingavat^1^, Wang Xinhui^1^, Liew Jia Min^1^, Farouq Bin Mahfut^2^, But Ka Pui^3^, Kok Yee Jiun^3^, Xuezhi Bi^3^, Yuansheng Yang^2^, Kobayashi Shintaro^4^, Tsoumpra Maria^4^ and Wei Zhang^1^*

^1^Downstream Processing Group, Bioprocessing Technology Institute, Agency for Science, Technology and Research (A*STAR), Singapore

^2^Cell Line Development Group, Bioprocessing Technology Institute, Agency for Science, Technology and Research (A*STAR), Singapore

^3^Protein Analytics Group, Bioprocessing Technology Institute, Agency for Science, Technology and Research (A*STAR), Singapore

^4^Chromatography Media Business Division, HOYA Technosurgical Corporation, Singapore Branch, Singapore

*Correspondence:

Wei Zhang, [zhang_wei@bti.a-star.edu.sg](mailto:zhang_wei@bti.a-star.edu.sg)


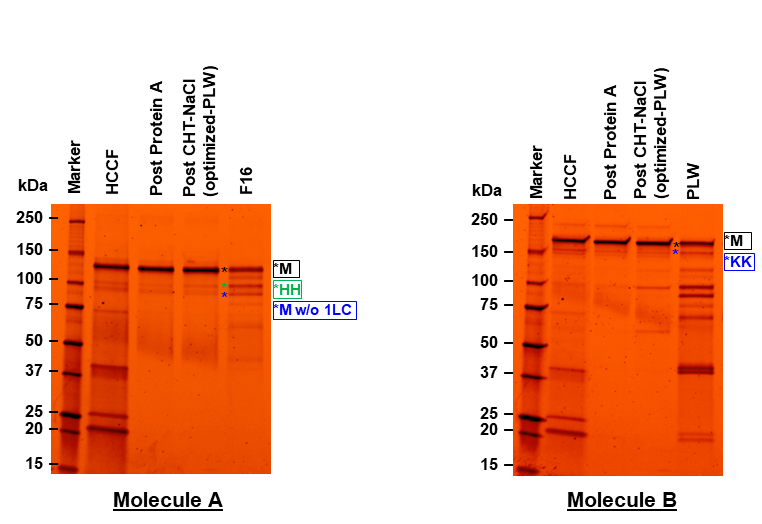


Figure S1. Non-reducing SDS-PAGEs, illustrating protein populations in HCCF, Post Protein A, Post CHT-P (optimized-PLW), and elution fractions before main product peaks (Molecule A: F16) or Molecule B: PLW fraction, of Molecule A and B, respectively. Marker units are in kDa. Protein populations, indicated as (*), were proposed as suggested by molecular weights, which were calculated from amino acid sequences. Abbreviations used are listed as followed; M = monomer; HH = hole-hole homodimer; KK = knob-knob homodimer; LC = light chain; w/o = without.


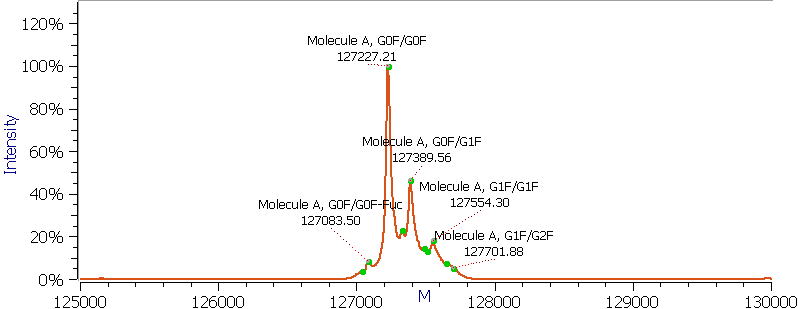

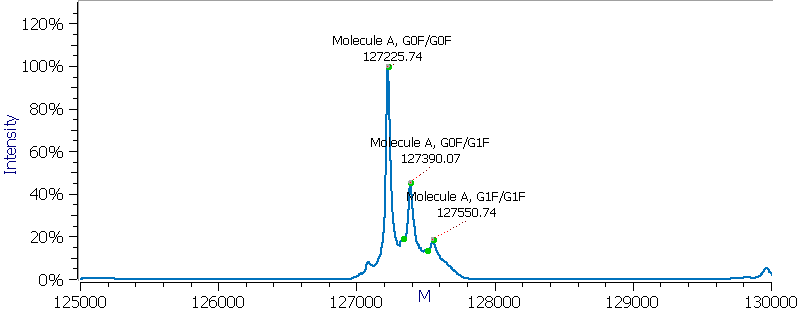

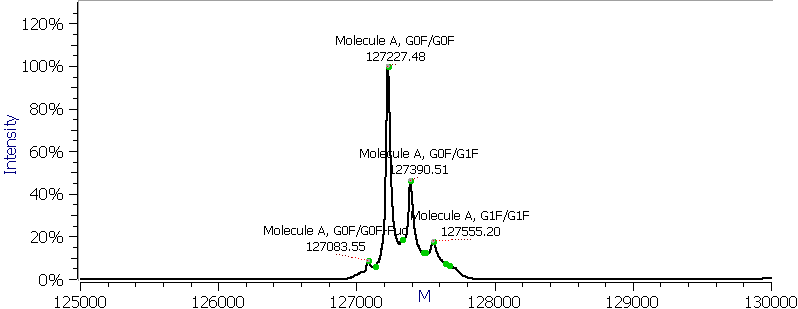

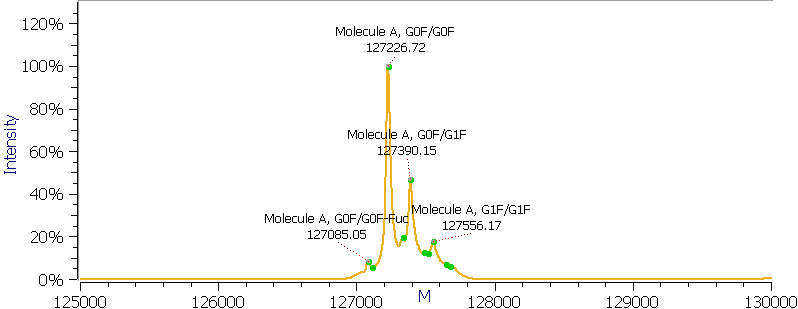


Post Protein A eluate

Post CEX product pool

Post CEX main elution peak

Post CHT-NaCl product pool with PLW optimization

Molecule A


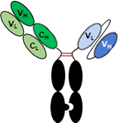


Figure S2. Intact mass analysis of Molecule A; post Protein A eluate; post CEX main elution peak; post CEX product pool; and post CHT-NaCl product pool with PLW optimization, respectively.

Molecule B


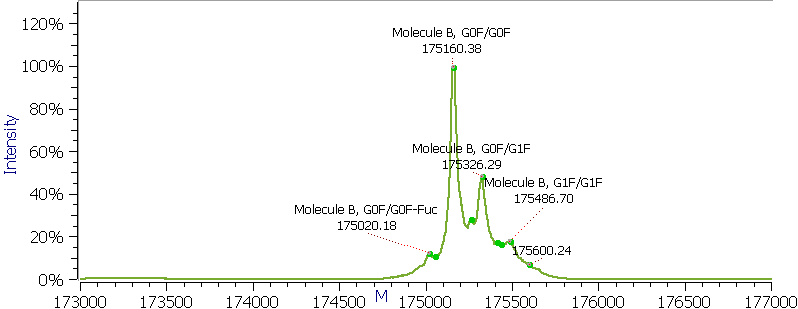

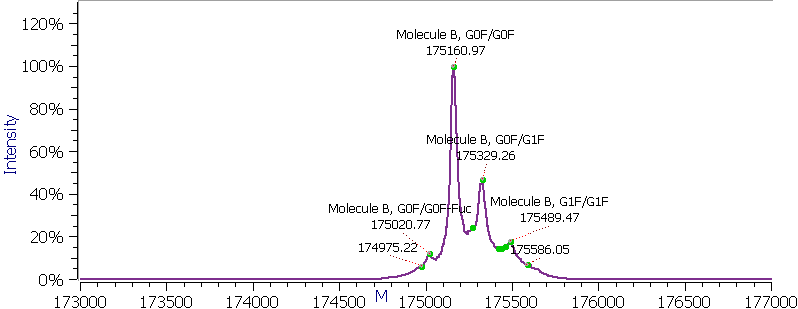

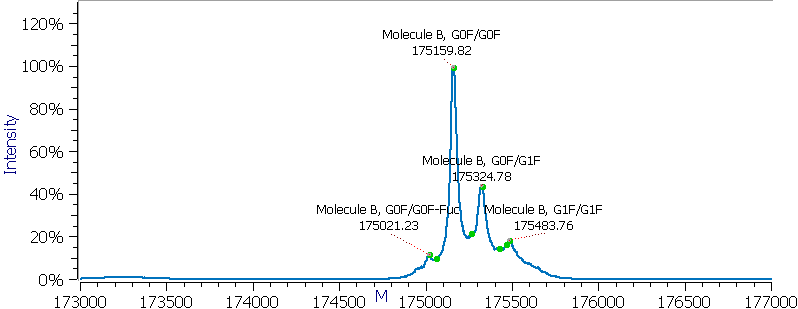

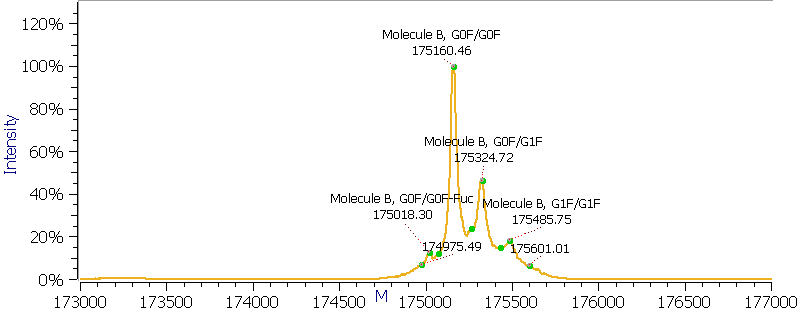


Post Protein A eluate

Post CEX product pool

Post CEX main elution peak

Post CHT-NaCl product pool with PLW optimization


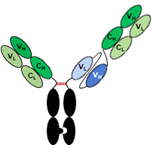


Figure S3. Intact mass analysis of Molecule B; post Protein A eluate; post CEX main elution peak; post CEX product pool; and post CHT-NaCl product pool with PLW optimization, respectively.


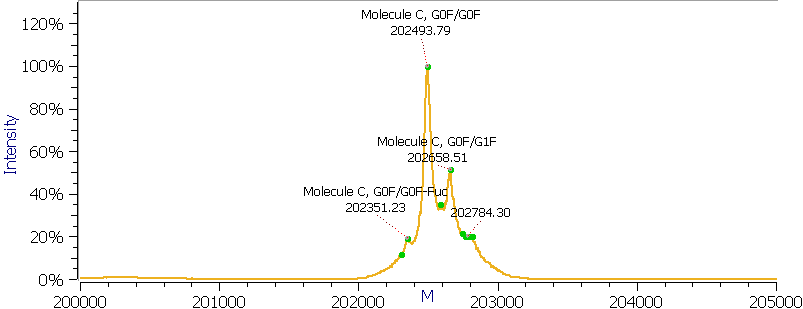

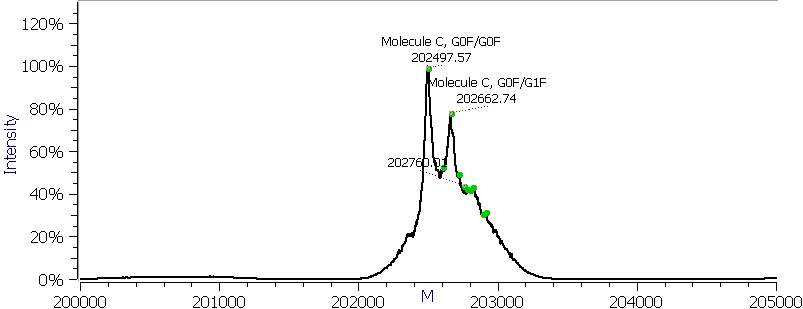

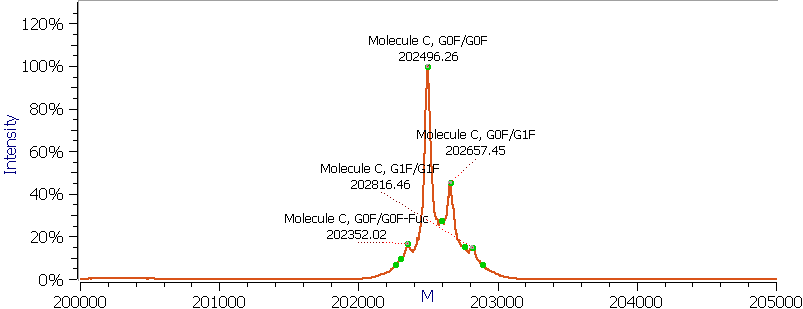

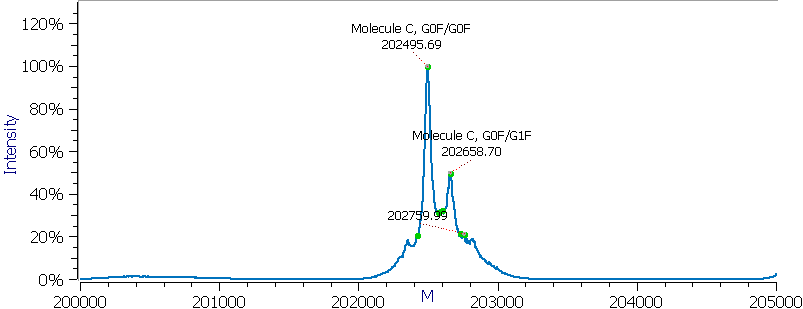

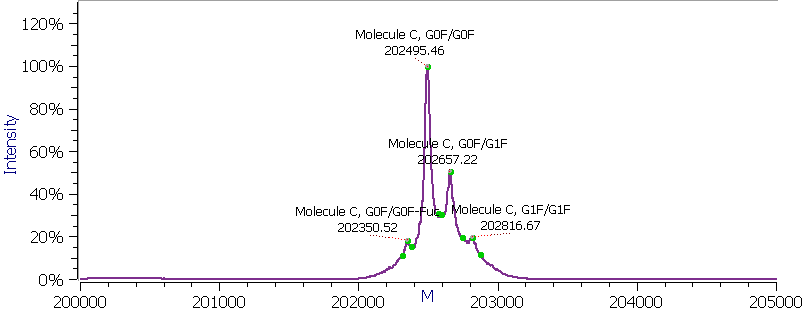


Post CEX main elution peak1

Post CHT-P product pool

Molecule C

Post CEX main elution peak2

Post Protein A eluate

Post CEX product pool


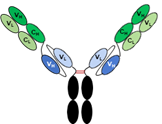


Figure S4. Intact mass analysis of Molecule C; post Protein A eluate; post CEX main elution peak1; post CEX main elution peak2; post CEX product pool; and post CHT-P product pool, respectively.


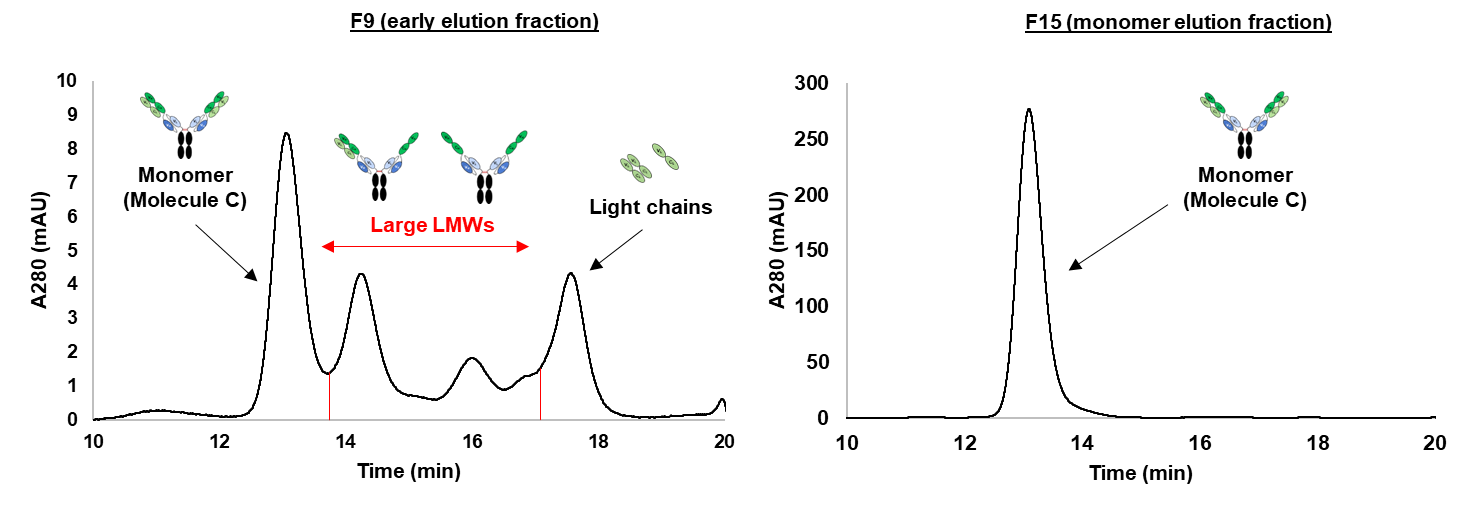


Figure S5. SEC-HPLC chromatograms of F9 (early elution fraction and F15 (monomer elution fraction) from a CHT-P run for the symmetric bsAb Molecule C. The chromatogram showed that CHT-P was able to remove large LMW species prior to elution of the target monomer.
